# Relevance of surveillance manual for the early detection of immune checkpoint inhibitor-induced myocarditis: A case series

**DOI:** 10.1016/j.apjon.2024.100598

**Published:** 2024-09-25

**Authors:** Takuya Oyakawa, Nao Muraoka, Kei Iida, Ayano Fujita, Koichi Yokoyama, Hiroshi Ishikawa, Haruyasu Murakami

**Affiliations:** a Division of Cardio-oncology, Shizuoka Cancer Center, Shizuoka, Japan; b Mishimatoukai Hospital, Shizuoka, Japan; c Nursing Department, Shizuoka Cancer Center, Shizuoka, Japan; d Department of Pharmacy, Shizuoka Cancer Center, Shizuoka, Japan; e Division of Advanced Medical Development, Shizuoka Cancer Center, Shizuoka, Japan

**Keywords:** Immune checkpoint inhibitor, Myocarditis, Surveillance, Troponin, Management flowchart, Cardio-oncology

## Abstract

**Objective:**

The European Cardio-Oncology Guidelines recommend regular electrocardiography and troponin testing during immune checkpoint inhibitors (ICIs) treatment, but their efficacy for monitoring ICI treatment remains unclear. This study aimed to evaluate the effectiveness of a surveillance protocol for early detection of ICI-induced myocarditis.

**Methods:**

Between May 2014 and May 2024, patients who began treatment with ICIs at our hospital and developed ICI-induced myocarditis were included in this study. We created a straightforward management flowchart for myocarditis. The protocol was based on monitoring troponin T levels. We confirmed the efficacy of our surveillance protocol using a case series of ICI-induced myocarditis.

**Results:**

During the observation period, 3481 patients were newly started on ICIs at our hospital. Five patients were previously diagnosed with myocarditis, and five patients were diagnosed with myocarditis after the implementation of the surveillance protocol. The manual enabled the early detection of myocarditis, and the mortality rate for myocarditis at our hospital improved from 60% to 0%. The incidence of conduction system disorders significantly reduced from 100% to 0% (*P* < 0.01). After the surveillance protocol was initiated, there were no cases of myocarditis requiring immunosuppressive drugs beyond steroids.

**Conclusions:**

This study confirmed the relevance of a troponin-based surveillance protocol for the early detection of ICI-induced myocarditis. The implementation of the surveillance protocol reduced mortality from myocarditis and significantly reduced serious complications of conduction system disorders. Although this study is a small case series of patients who developed myocarditis, we confirm the effectiveness of surveillance for myocarditis.

## Introduction

Immune checkpoint inhibitors (ICIs) cause various immune-related adverse events,^1^ including myocarditis. Myocarditis caused by ICIs is rare; however, if the diagnosis is delayed, the prognosis is poor, and the mortality rate is > 40%.^2^^,^^3^ Although the early detection and diagnosis of ICI-induced myocarditis are required, a clear strategy for monitoring the early detection of myocarditis is not available. Patients who develop ICI-induced myocarditis have high rates of electrocardiography (ECG) and/or blood tests for troponin abnormalities.^2^ For this reason, although there is no evidence, the European Cardio-Oncology Guidelines suggest that before starting ICIs, ECG results, troponin and brain natriuretic peptide levels, and echocardiogram findings should be checked according to cardiovascular risk and that ECG and troponin examination should be regularly performed during treatment.^4^ However, the usefulness of this monitoring method remains unclear. Routine ECG and troponin testing have not been performed in the clinical trials of ICIs. Clinical trials examine only ECG results before initiating ICI treatment, regardless of cardiovascular risk.^5^ For these reasons, some physicians, laboratory technicians, and clinical nurses believe that it is difficult to regularly perform complex cardiovascular risk assessments and multiple tests because myocarditis has an incidence rate of < 1%.^6^ Therefore, we created a myocarditis surveillance protocol that can be implemented in all patients at our hospital, where ICIs are used approximately 500 times monthly. Our hospital's Cancer Immunotherapy Management Committee approved this protocol, which was put into practice in January 2023. We aimed to confirm the efficacy of our surveillance protocol using a case series of ICI-induced myocarditis.

## Methods

### Monitoring protocol

**Fig. 1:**
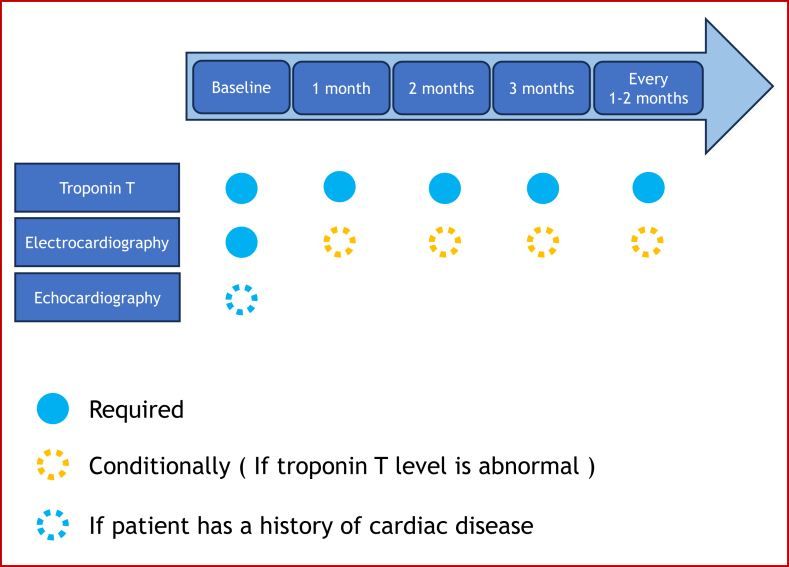
Overview of monitoring.

**Fig. 2:**
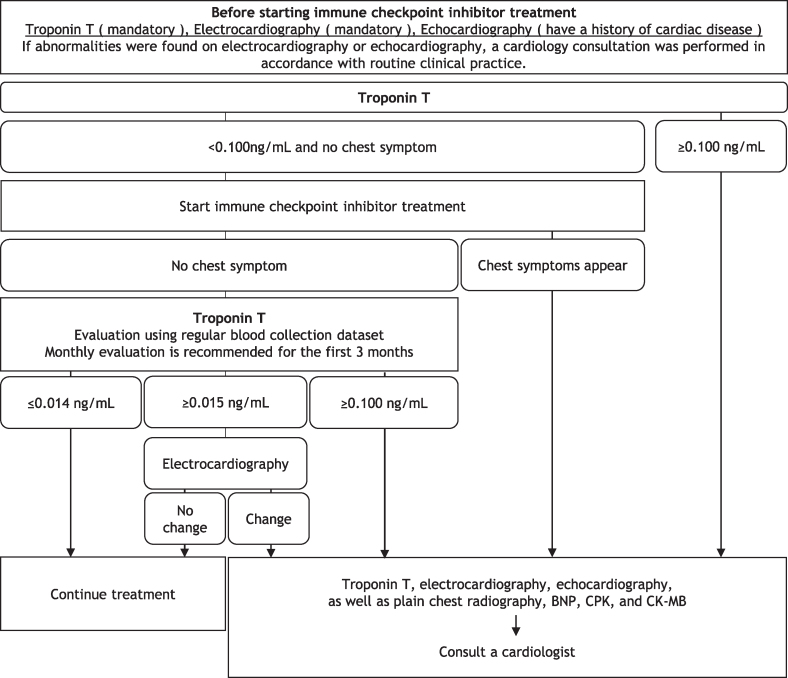
Myocarditis (cardiovascular adverse event) management manual (version 1.1). Additional circulatory system tests or consultations for cardiologist are required when: 1) Troponin level is ≥ 0.100 ng/mL; 2) Chest symptoms appear; 3) Troponin level is ≥ 0.015 ng/mL and there are electrocardiography changes. BNP, B-type natriuretic peptide; CPK, creatinine phosphokinase; CK-MB, creatine kinase-MB isoenzyme.

The protocol was based on monitoring with the addition of troponin T to routine blood tests performed during cancer treatment (Fig. 1). Patients with a history of cardiac disease should undergo echocardiography before initiating ICI therapy. ECG should be performed before initiating ICI; however, after ICI initiation, ECG should be performed only if troponin T levels are abnormal. The management flowchart is shown in Fig. 2.

The monitoring protocol has the following steps:

1) Troponin T levels and ECG results are assessed before starting ICI treatment. If a patient has a history of cardiac disease, echocardiography results are also assessed.
2) Patients are monitored for chest symptoms and troponin T levels after ICI initiation.
3) If chest symptoms appear after starting ICIs, the specified examinations are performed, and the patients consult a cardiologist.
4) If the troponin T level is normal (≤ 0.014 ng/mL), ICI treatment is continued.
5) An additional ECG should be performed if the troponin T level is abnormal (≥ 0.015 ng/mL). If the ECG result is normal or there are no changes, ICI is continued; if the ECG result is abnormal or there are changes, the patients consult a cardiologist.
6) The patients consult a cardiologist if the troponin T level is within a panic value (≥ 0.100 ng/mL).

This manual monitors troponin T levels using blood tests. Troponin T levels are added to the ICI blood collection dataset. If the troponin T value is abnormal, it is highlighted in red, and an ECG is performed. To facilitate the assessment, a troponin T value of ≥ 0.100 ng/mL is considered a panic value, highlighted in green, and reported to the requesting physician. Troponin level is naturally elevated in 5% of patients and shows abnormal values without pathological significance.^7^ Therefore, troponin T values were classified as abnormal or panicked. A troponin T level of 0.100 ng/mL is also the cutoff value for myocardial infarction.^8^ In patients with ICI myocarditis, the troponin T value almost exceeds 0.100 ng/mL.^9^

### Data analysis

Between May 2014 and May 2024, patients who newly started ICIs at our hospital and developed ICI-induced myocarditis were included in this study. The clinical course of myocarditis before and after the implementation of the surveillance protocol was identified and examined.

Data were collected on age, sex, type of cancer, type of ICI, time to myocarditis, concomitant immune-related adverse events, grade of myocarditis at the time of diagnosis (Common Terminology Criteria for Adverse Events version 5.0), chest symptoms (chest pain, palpitation, dyspnea, fatigue, breathlessness, dizziness, presyncope, syncope) at the time of diagnosis, troponin levels at diagnosis, creatinine phosphokinase levels at diagnosis, ECG changes from baseline to diagnosis, course of myocarditis, treatment of myocarditis, outcome, and time from myocarditis. The factors related to the clinical course were statistically analyzed.

Two-group comparisons were performed using Fisher's exact test. Data were analyzed using JMP 9 (SAS Institute Inc., Cary, NC, USA), and *P* < 0.05 was considered statistically significant.

### Ethical considerations

This study was approved by our institutional review board (IRB No. J2024-7-2024-1-3). Informed consent was obtained using the opt-out methodology. All procedures involving human participants performed in this study were in accordance with the ethical standards of the institutional and/or national research committee and with the 1964 Declaration of Helsinki and its later amendments or comparable ethical standards.

## Results

During the observation period, 3481 patients received ICI treatment at our hospital. Ten patients developed new ICI-induced myocarditis. The incidence rate of ICI-induced myocarditis at our hospital was 0.3%. Five patients were previously diagnosed with myocarditis, and five patients were diagnosed with myocarditis after the implementation of the surveillance protocol.

The patient characteristics and clinical courses are shown in Table 1. The median troponin levels at diagnosis were 1.515 ng/mL before the protocol implementation and 0.241 ng/mL after the implementation. Regarding ECG changes, conduction system disorders, such as sick sinus syndrome and right or left bundle branch block, they no longer appeared after the protocol was implemented. Before the implementation of the protocol, the course of myocarditis often led to severe and sudden worsening of symptoms, such as sick sinus syndrome, complete atrioventricular block, conduction system disorders, ventricular tachycardia, and cardiac tamponade. However, after introducing the protocol, most of the symptoms, such as decreased left ventricular ejection fraction and myocardial edema, were relatively mild. Before the implementation of the protocol, the incidence rate of conduction system disorders was 100%; after the implementation, the incidence rate was 0%. The incidence rate of conduction system disorders was also significantly lower (*P* < 0.01). The mortality rate due to myocarditis improved from 60% to 0% with the introduction of the protocol (*P* = 0.17) (Table 2). After the surveillance protocol was initiated, there were no cases of myocarditis requiring immunosuppressive drugs beyond steroids. When chest symptoms were considered, including fatigue, breathlessness, and dyspnea, all patients had chest-related symptoms.

**Table 1:** Clinical course of patients with immune checkpoint inhibitor-induced myocarditis.

Patients	Manual monitoring	Age (years)	Sex	Types of cancer	Types of immune checkpoint inhibitors	Time to onset of myocarditis (days)	Concomitant irAE	Grade of myocarditis at time of diagnosis	Chest symptoms at time of diagnosis^a^	Troponin T value (ng/mL) at time of diagnosis	CPK (U/L) at time of diagnosis	Electrocardiography change from baseline at time of diagnosis	Clinical course of myocarditis	Treatment of myocarditis	Outcome of myocarditis	Time to outcome (days)
1	No	81	Female	Lung	Nivolumab	24	–	G4	Yes	0.081	49	Left posterior hemiblock. Sick sinus syndrome Atrial fibrillation (Right bundle branch block from baseline)	Sick sinus syndrome Cardiac tamponade Atrial fibrillation	Steroid pulse Predonine Pericardial drainage Temporary pacing Implantation of a pacemaker	Alive	–
2	No	66	Female	Lung	Pembrolizumab	27	Myositis Hepatic disorder	G3	Yes	–	9744	ST-T wave change ST segment elevation Negative T wave Right bundle branch block	Sick sinus syndrome	Predonine Mycophenolate mofetil	Death	1
3	No	73	Female	Lung	Nivolumab	23	Myositis Myasthenia gravis Erythroderma	G3	Yes	4.500	5605	Right bundle branch block ST-T wave change ST segment elevation Negative T wave ST depression	Complete atrioventricular block Sick sinus syndrome Ventricular tachycardia	Steroid pulse Methylprednisolone infliximab	Alive	–
4	No	77	Male	Bladder	Pembrolizumab	34	Myositis	G3	Yes	1.720	881	ST-T wave change (Left bundle branch block from baseline)	Heart failure Ventricular tachycardia Completely atrioventricular block Sick sinus syndrome	Steroid pulse Methylprednisolone	Death	17
5	No	67	Female	Malignant melanoma	Nivolumab	27	Hepatic disorder	G3	Yes	1.310	3308	Right bundle branch block Left anterior hemiblock ST-T wave change ST segment elevation Negative T wave	Sick sinus syndrome	Steroid pulse Predonine	Death	23
6	Yes	67	Male	Hepatocellular	Atezolizumab	38	Thyroid deficiency	G3	Yes	0.241	50	ST-T wave change Negative T wave	Ventricular tachycardia	Predonine	Alive	–
7	Yes	25	Female	Breast	Pembrolizumab	20	–	G3	Yes	0.171	63	ST-T wave change ST segment elevation Negative T wave	Reduced left ventricular ejection fraction Myocardial edema	Steroid pulse Predonine	Alive	–
8	Yes	45	Female	Breast	Pembrolizumab	24	–	G3	Yes	0.748	230	ST-T wave change Negative T wave ST segment elevation	Reduced left ventricular ejection fraction Myocardial hypertrophy	Steroid pulse Predonine	Alive	–
9	Yes	65	Male	Gastric	Nivolumab	28	Thyroid deficiency	G2	Yes	0.099	42	ST-T wave change Negative T wave Atrial fibrillation	Reduced left ventricular ejection fraction Heart failure Atrial fibrillation	Predonine	Alive	–
10	Yes	76	Male	Pharyngeal	Nivolumab	2525	Hepatic disorder Pneumonitis SIADH	G2	Yes	0.403	383	ST-T wave change Negative T wave	Heart failure Myocardial hypertrophy	Methylprednisolone	Alive	–

irAE, immune-related adverse event; CPK, creatinine phosphokinase; SIADH, syndrome of inappropriate antidiuretic hormone secretion.

a Chest symptoms include fatigue, breathlessness, dyspnea, chest pain, palpitations, dizziness, and syncope.

**Table 2:** Changes after introducing the management manual.

	After the start of the management manual	Before the start of the management manual	RR (95% CI)	*P*-value
Death caused by myocarditis	0%	60%	–	0.17
Impulse conducting system disorder	0%	100%	–	< 0.01

RR, risk ratio.

## Discussion

This study confirmed the relevance of a troponin-based surveillance protocol for the early detection of ICI-induced myocarditis. The implementation of the surveillance protocol reduced mortality from myocarditis and markedly reduced serious complications of conduction system disorders. Although this study involves a small case series of patients who developed myocarditis, it is the first to confirm the effectiveness of surveillance for myocarditis.

### Surveillance protocols

Adverse cardiovascular events associated with ICIs include myocarditis, conduction disease, pericarditis, coronary artery disease, and noninflammatory left ventricular dysfunction.^10^ Myocarditis is an inflammatory disease of the myocardium, encompassing several diseases that include several pathological conditions. Myocarditis may present with a wide range of symptoms, including mild dyspnea or chest pain, which resolve without specific therapy for cardiogenic shock, life-threatening arrhythmia, and death.^11^ Myocarditis has a low incidence rate, but once it occurs, the mortality rate is as high as 40% to 50%.^2^ Early detection is important, and surveillance protocols have been suggested in European guidelines.^4^ Troponin level assessment or ECG is considered the most helpful method for diagnosing ICI-induced myocarditis.^2^ Therefore, we referred to the European guidelines and created a monitoring protocol based on troponin levels that can be implemented at our hospital. No previous studies have examined the efficacy of surveillance protocols for the early diagnosis of ICI myocarditis. Although this is a small case series, it is the first study to confirm the usefulness of a troponin-based surveillance protocol. The use of a surveillance protocol has reduced the mortality rate associated with myocarditis. In addition, the incidence rate of fatal conduction system disorders has considerably reduced. Seven patients survived after myocarditis resolved, but two survivors who were treated prior to the implementation of the surveillance protocol required emergency temporary cardiac pacing, pacemaker implantation, and pericardial drainage and were admitted to the intensive care unit with unstable vital signs and life-threatening arrhythmias. After the implementation of the monitoring protocol, the survivors did not require any special treatment, improved with steroids alone, and could be treated in a general ward. Therefore, surveillance protocols may be useful for the management of myocarditis.

### ECG findings

ECG findings of a widened QRS complex, left bundle branch block, prolonged QTc, advanced atrioventricular block, and sustained ventricular tachycardia indicate poor prognosis in patients with myocarditis.^12^, ^13^, ^14^, ^15^, ^16^, ^17^ In contrast, an ECG showing no abnormalities or ST elevation similar to that observed in pericarditis suggests a good prognosis for myocarditis.^18^ Patients who develop atrioventricular block due to myocarditis often develop bundle branch block.^19^ Regarding conduction system disorders caused by myocarditis, as myocarditis progresses, the bundle branch block deteriorates to an atrioventricular block.^20^ For this reason, the appearance of bundle branch block is thought to be a precursor to the progression of conduction system disorders, such as atrioventricular block. Before monitoring using the management protocol, bundle branch block developed in four of the five patients. The remaining patient initially had a left bundle branch block. All five patients developed atrioventricular block or sick sinus syndrome. If a bundle branch block appears in ICI-induced myocarditis, it may be a precursor to atrioventricular block or sick sinus syndrome. In addition, patients with bundle branch block may be more susceptible to conduction system disorders due to ICI-induced myocarditis.

### Treatment

Steroids are used to treat ICI-induced myocarditis, but in severe cases, additionally, non-steroidal immunosuppressive agents are also used.^21^ Corticosteroids serve as the first-line treatment for ICI-induced myocarditis. Although there is limited data on second-line treatments, options such as tacrolimus, mycophenolate mofetil, infliximab, and antithymocyte globulin are considered.^22^ Other potential treatments include abatacept, tofacitinib, tocilizumab, alemtuzumab, and plasma exchange.^4^ Before implementing the surveillance protocol, two of the five patients were prescribed immunosuppressive agents other than steroids; however, after implementing the surveillance protocol, no patients received non-steroidal immunosuppressive agents. This suggests that the introduction of a surveillance protocol allows the diagnosis of ICI-induced myocarditis before it becomes severe. This surveillance protocol may reduce the incidence of ICI-induced myocarditis, which requires immunosuppressive medications other than steroids.

### Troponin

Troponin T levels at the time of ICI-induced myocarditis were higher before the implementation of the surveillance protocol than after its implementation. Among patients whose troponin T level was measured after the implementation of the surveillance protocol, the troponin T value at the time of diagnosis was ≤ 1 ng/mL. However, three patients had a troponin T value of ≥ 1 ng/mL before management using the surveillance protocol. Before the implementation of the surveillance protocol, some patients had troponin T values that were one order of magnitude higher at the time of diagnosis than after the implementation of the surveillance protocol. Troponin levels are sensitive indicators of myocardial injury.^23^ After initiating the surveillance protocol, troponin levels at diagnosis were low, suggesting that myocarditis can be diagnosed at a mild stage.

### Clinical setting

When manual monitoring is not performed, for example, when blood sampling for troponin T or ECG is not performed, it is important to check with the patient's physician. This can be observed not only by a doctor but also by a nurse or pharmacist at the outpatient chemotherapy center or ward and during the patient's doctor checks. At our hospital, nurses and pharmacists also consult with the patient's doctor. This reduces the deviations from the monitoring protocol.

### Limitations

This study has some limitations. First, it was a case series involving a few patients who developed myocarditis. As it only focused on these specific patients, we cannot make definitive statements about the overall effectiveness of this protocol. Future studies are required to include all patients who underwent troponin measurements. Second, this monitoring protocol relies on troponin measurements. Although early detection of myocarditis has been achieved by combining chest symptoms with ECG changes, the simplicity of this approach might limit its effectiveness in patients without elevated troponin levels. Therefore, additional biomarkers may be necessary for comprehensive detection. Third, we could not evaluate whether this manual caused delays or postponements in ICI treatment or decreased the efficacy of cancer treatment. Furthermore, not all patients were managed according to our hospital's manual.

## Conclusions

**Fig. 3:**
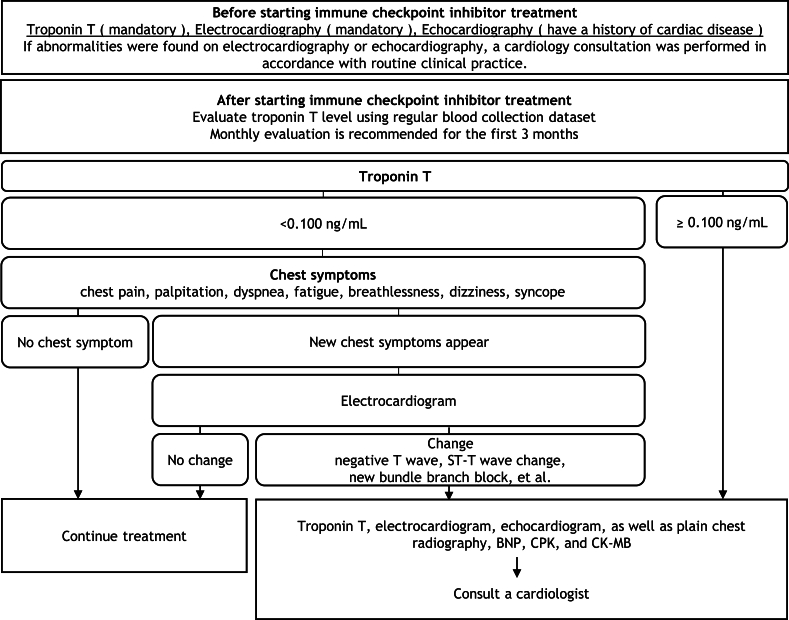
Myocarditis management manual (version 2.0). Additional circulatory system tests or consultations for cardiologist are required when: 1) Troponin level is ≥ 0.100 ng/mL. Other provisions in the manual are the same as those in daily clinical practice. BNP, B-type natriuretic peptide; CPK, creatinine phosphokinase; CK-MB, creatine kinase-MB isoenzyme.

In this study, we confirmed the effectiveness of a monitoring protocol for the early detection of ICI-related myocarditis. It is believed that the frequency of ICI use will continue to increase in the future, and the incidence rate of myocarditis is expected to increase. Simple monitoring protocols are easier to implement. Having confirmed the effectiveness of this monitoring protocol, monitoring may be possible using simpler methods. Therefore, we introduced a revised monitoring protocol (Fig. 3). In the future, we aim to confirm the effectiveness of the revised monitoring protocol.

## Ethics statement

This study was approved by our institutional review board (IRB No. J2024-7-2024-1-3). Informed consent was obtained using the opt-out methodology.

## Funding

This study received no external funding.

## CRediT authorship contribution statement

**Takuya Oyakawa:** Conceptualization, Methodology, Data curation, Formal analysis, Writing – Original draft preparation. **Nao Muraoka:** Writing – review and editing. **Kei Iida:** Writing – review and editing. **Ayano Fujita:** Writing – review and editing. **Yokoyama Koichi:** Writing – review and editing. **Ishikawa Hiroshi:** Writing – review and editing. **Murakami Haruyasu:** Writing – review and editing. All authors had full access to all the data in the study, and the corresponding author had final responsibility for the decision to submit for publication. The corresponding author attests that all listed authors meet authorship criteria and that no others meeting the criteria have been omitted.

## Data availability statement

Data availability does not apply to this article, as no new data were created or analyzed in this study.

## Declaration of generative AI and AI-assisted technologies in the writing process

No AI tools/services were used during the preparation of this work.

## Declaration of competing interest

Takuya Oyakawa received honoraria from Pfizer, Daiichi Sankyo, TOA EIYO, Novartis, AstraZeneca, and Bristol-Myers Squibb. Nao Muraoka has no conflict of interest. Kei Iida has no conflict of interest. Ayano Fujita has no conflict of interest. Yokoyama Koichi has no conflict of interest. Ishikawa Hiroshi has no conflict of interest. Haruyasu Murakami reports grants from Chugaipharma, AstraZeneca, Abbvie, Daiichi Sankyo, IQvia, and Taiho Pharmaceutical, Bayer, and received honoraria from Chugai pharma, Daiichi Sankyo, AtraZeneca, Takeda, Amgen, Ono Pharmaceutical, Bristol-Myers Squibb Japan, MSD, Pfizer, Novartis Lilly Japan, Taiho pharmaceutical, and Eisai, Nihonkayaku, as well as participation on a data safety monitoring board or advisory board, outside the submitted work.

## References

[1] Tan S., Day D., Nicholls S.J., Segelov E. (2022). Immune checkpoint inhibitor therapy in oncology: current uses and future directions: *JACC: CardioOncology* state-of-the-art review. JACC CardioOncol.

[2] Mahmood S.S., Fradley M.G., Cohen J.V. (2018). Myocarditis in patients treated with immune checkpoint inhibitors. J Am Coll Cardiol.

[3] Salem J.E., Manouchehri A., Moey M. (2018). Cardiovascular toxicities associated with immune checkpoint inhibitors: an observational, retrospective, pharmacovigilance study. Lancet Oncol.

[4] Lyon A.R., López-Fernández T., Couch L.S. (2022). 2022 ESC guidelines on cardio-oncology developed in collaboration with the European Hematology association (EHA), the European Society for Therapeutic Radiology and oncology (ESTRO) and the International Cardio-oncology Society (IC-OS). Eur Heart J.

[5] Hellmann M.D., Paz-Ares L., Bernabe Caro R. (2019). Nivolumab plus ipilimumab in advanced non-small-cell lung cancer. N Engl J Med.

[6] Moslehi J.J., Johnson D.B., Sosman J.A. (2017). Myocarditis with immune checkpoint blockade. N Engl J Med.

[7] Mariathas M., Allan R., Ramamoorthy S. (2019). True 99th centile of high sensitivity cardiac troponin for hospital patients: prospective, observational cohort study. BMJ.

[8] Wu A.H., Valdes R., Apple F.S. (1994). Cardiac troponin-T immunoassay for diagnosis of acute myocardial infarction. Clin Chem..

[9] Vasbinder A., Chen Y., Procureur A. (2022). Biomarker trends, incidence, and outcomes of immune checkpoint inhibitor-induced myocarditis. JACC CardioOncol.

[10] Lyon A.R., Yousaf N., Battisti N.M.L., Moslehi J., Larkin J. (2018). Immune checkpoint inhibitors and cardiovascular toxicity. Lancet Oncol.

[11] Cooper L.T. (2009). Myocarditis. N Engl J Med.

[12] Ukena C., Mahfoud F., Kindermann I., Kandolf R., Kindermann M., Böhm M. (2011). Prognostic electrocardiographic parameters in patients with suspected myocarditis. Eur J Heart Fail.

[13] Ammirati E., Veronese G., Brambatti M. (2019). Fulminant versus acute nonfulminant myocarditis in patients with left ventricular systolic dysfunction. J Am Coll Cardiol.

[14] Magnani J.W., Danik H.J., Dec G.W., DiSalvo T.G. (2006). Survival in biopsy-proven myocarditis: a long-term retrospective analysis of the histopathologic, clinical, and hemodynamic predictors. Am Heart J.

[15] Nakashima H., Katayama T., Ishizaki M., Takeno M., Honda Y., Yano K. (1998). Q wave and non-Q wave myocarditis with special reference to clinical significance. Jpn Heart J.

[16] Ogunbayo G.O., Elayi S.C., Ha L.D. (2019). Outcomes of heart block in myocarditis: a review of 31,760 patients. Heart Lung Circ.

[17] Adegbala O., Olagoke O., Akintoye E. (2019). Predictors, burden, and the impact of arrhythmia on patients admitted for acute myocarditis. Am J Cardiol.

[18] Buttà C., Zappia L., Laterra G., Roberto M. (2020). Diagnostic and prognostic role of electrocardiogram in acute myocarditis: a comprehensive review. Ann Noninvasive Electrocardiol.

[19] Nakashima H., Honda Y., Katayama T. (1994). Serial electrocardiographic findings in acute myocarditis. Intern Med.

[20] Elizari M.V., Chiale P.A. (1993). Cardiac arrhythmias in Chagas' heart disease. J Cardiovasc Electrophysiol.

[21] Schneider B.J., Naidoo J., Santomasso B.D. (2021). Management of immune-related adverse events in patients treated with immune checkpoint inhibitor therapy: ASCO guideline update. J Clin Oncol.

[22] Waliany S., Lee D., Witteles R.M. (2021). Immune checkpoint inhibitor cardiotoxicity: understanding basic mechanisms and clinical characteristics and finding a cure. Annu Rev Pharmacol Toxicol.

[23] Lauer B., Niederau C., Kühl U. (1997). Cardiac troponin T in patients with clinically suspected myocarditis. J Am Coll Cardiol.

